# Application of a Video Head Impulse Test in the Diagnosis of Vestibular Neuritis

**DOI:** 10.3390/life14060757

**Published:** 2024-06-13

**Authors:** Agnieszka Jasinska-Nowacka, Kazimierz Niemczyk

**Affiliations:** Department of Otorhinolaryngology, Head and Neck Surgery, Medical University of Warsaw, 02-091 Warszawa, Poland; kazimierz.niemczyk@wum.edu.pl

**Keywords:** vestibular neuritis, acute unilateral vestibulopathy, vertigo, vestibulo-ocular reflex, vHIT

## Abstract

In patients presenting in the emergency department with acute vertigo, a rapid and accurate differential diagnosis is crucial, as posterior circulation strokes can mimic acute vestibular losses, leading to inappropriate treatment. The diagnosis of vestibular neuritis is made based on the clinical manifestation and a bedside otoneurological assessment. In the clinical examination, an evaluation of the vestibulo-ocular reflex is the key element; however, the accuracy of the bedside head impulse test depends on the clinician’s experience. Thus, new diagnostic methods are needed to objectify and facilitate such rapid vestibular evaluations. The aim of our paper is to provide a comprehensive review of the video head impulse test’s application in the diagnosis of vestibular neuritis. Numerous studies have reported advantages that make this method helpful in detailed otoneurological evaluations; in contrast to the bedside head impulse test, it enables an analysis of all six semicircular canals function and records the covert corrective saccades, which are invisible to the naked eye. As a portable and easy diagnostic tool, it is known to improve the diagnostic accuracy in patients with acute vertigo presenting in the emergency department. Moreover, as it evaluates the vestibulo-ocular reflex across different frequencies, as compared to caloric tests, it can be used as an additional test that is complementary to videonystagmography. Recently, several papers have described the application of the video head impulse test in follow-up and recovery evaluations in patients with vestibular neuritis.

## 1. Introduction

Vestibular neuritis (VN) is defined as an acute loss of peripheral vestibular function without audiological symptoms or signs. The clinical manifestation is characterized by an episode of spinning vertigo accompanied by nausea and vomiting lasting several hours or days; however, balance problems may last longer [1,2,3]. Despite numerous studies having been performed, the etiology remains not fully understood, with inflammatory (mainly the reactivation of the latent herpes simplex type 1 infection), autoimmune and vascular factors taken into consideration [4]. In consequence, according to the consensus document established by the Barany Society [5], the term acute unilateral vestibulopathy is proposed as a synonym of VN, as it better reflects the nature of the disease.

### Clinical Manifestation and Differential Diagnosis of VN

The diagnosis of VN is based on the clinical manifestation and an otoneurological examination [5]. The characteristic symptoms involves spinning vertigo attacks with acute onset accompanied by intensive nausea, vomiting and balance problems [1,2,4,5]. 

Typically, VN manifests as the first vertigo attack in the patient’s life and has to be differentiated from labyrinthitis, characterized by an acute spinning episode accompanied by sudden unilateral hearing loss [6]. However, in the differential diagnosis, the first attacks of other peripheral vestibulopathies have to be taken into consideration as well. First, the common causes of spinning vertigo episode are vestibular migraine and Meniere’s disease [7,8]. In Meniere’s disease episodes, hearing loss and tinnitus are the distinguishing features, although 50% of patients do not present the full clinical manifestation in the early stage of the disease [9]. Additionally, benign paroxysmal positional vertigo (BPPV), particularly affecting the lateral semicircular canal, has to be excluded, as it can manifest with pseudo-spontaneous horizontal nystagmus [10].

In the emergency department, benign peripheral vertigo must be differentiated from central pathologies. Unfortunately, several serious pathologies that require immediate treatment may be misdiagnosed in the emergency department, as they can mimic VN. Despite the typical clinical manifestation, 6–39% of patients diagnosed as having peripheral vestibular loss can possibly suffer from central pathologies, i.e., posterior fossa stroke [11,12,13,14]. Thus, a fast and sensitive diagnostic method is needed to evaluate and objectify the vestibular function in patients with acute vertigo.

## 2. Otoneurological Examination

### 2.1. HINTS Protocol

The role of an otoneurological examination is to confirm a unilateral vestibular deficit and to exclude central neurological pathologies. In patients with an acute vertigo episode with nystagmus, a HINTS (head impulse nystagmus test of skew) examination is recommended to quickly evaluate the probability of central vestibular pathologies. The protocol includes three elements: a head impulse test (HIT), assessing the horizontal vestibulo-ocular reflex (VOR); a nystagmus evaluation; a test of skew (cover test) [15,16]. In patients with acute vestibular loss, a characteristic pattern of signs is present, namely corrective saccades indicating VOR impairment (visible in the HIT during head movement toward the side of lesion), horizontal or horizontal–torsional nystagmus with fast faze beating opposite to the affected side, and no vertical divergence in the alternating cover test. Any discrepancies in the response pattern described above suggest a central cause of acute vertigo and require immediate neurological diagnostics. The HINTS examination lasts only a few minutes and can be performed in the emergency department. It was reported that the protocol has 100% sensitivity and 96% specificity for detecting a stroke, constituting a better result than diffusion-weighted magnetic resonance imaging (DWI MRI), characterized by 12% false negative results if performed within the first 48 h after symptom onset [15]. Nevertheless, to achieve that level, a trained otoneurologist performing a HINTS is needed in the emergency department [17].

In 2013, Newman-Toker et al. [18] described a modification of the HINTS protocol with an additional hearing evaluation (finger rub test). The HINST-plus protocol is known to have higher diagnostic sensitivity (99%) for stroke cases and can be used in rapid clinical evaluations. Moreover, as the cochlea is supplied by the anterior inferior cerebellar artery (AICA) in 80% of the population, hearing loss is more likely to occur in patients with AICA strokes in comparison to cases of posterior inferior cerebellar artery (PICA) infarction [19].

### 2.2. VOR Evaluation in Unilateral Vestibular Loss

The VOR assessment was first described by Curthoys and Halmagyi in 1988 [20], and since then it has become a necessary part of otoneurological examinations. To record and measure the reflex, the authors used a scleral search coil with contact lenses used by the patients to prove that the HIT examination can evaluate the function of the lateral semicircular canal.

**Figure 1:**
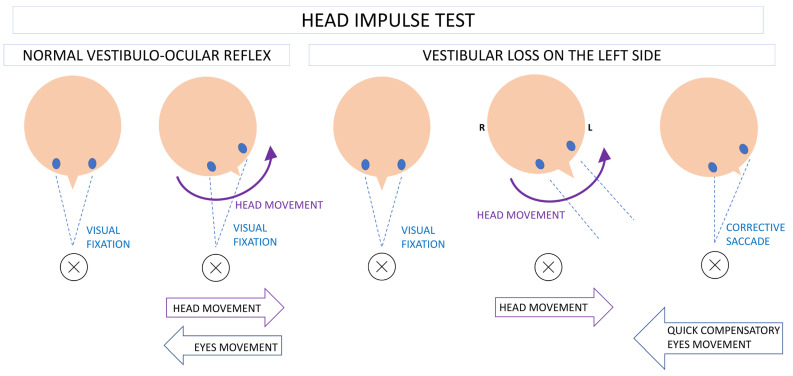
Horizontal vestibulo-ocular reflex (VOR) evaluation using a head impulse test (HIT). Normal HIT—head movement to the left results in movement of the eyes in opposite direction. Abnormal HIT in a patient with vestibular loss affecting the lateral semicircular canal on the left side; despite head movement, the VOR cannot be activated, which causes loss of fixation. Then, a corrective saccade of a central origin enables refixation of the visual target.

The bedside VOR evaluation is performed by applying the head thrust test (head impulse test—HIT) (Figure 1). During the examination, the patient sits in front of a doctor and is instructed to focus on the examiner’s nose. The physician performs vigorous movements of the patient’s head, made in a horizontal plane, with an appropriate speed, as the reflex has a latency of 7 to 10 ms. Thus, if the head movements are too slow or prolonged, saccades and smooth pursuit, which are movements of a central origin, will also contribute to maintaining focus on the target. Secondly, the movement direction should be random to avoid anticipatory eye movements in cases of alternating predictable trials toward the right and left side. While turning the head to the left, a normal angular horizontal VOR results in the movement of the eyes to the right (Figure 1). The speed of the eyes’ movement is close to the speed of head movement, although the direction is opposite to allow the image to be projected onto the central fovea of the retina and to maintain dynamic visual acuity. In patients with vestibular loss, after moving the head towards the affected side, the patient’s eyes will unconsciously follow the head because the proper vestibulo-ocular reflex cannot be activated. Then, a quick corrective saccade of central origin will enable the patient’s gaze to refocus on the given target.

## 3. Video Head Impulse Test

Nowadays, the HIT is a key examination that allows for the rapid evaluation of the vestibular function. Nevertheless, a bedside VOR assessment is subjective, with the sensitivity of the test depending on the examiner’s experience [16,17]. Thus, new technologies enabling the VOR evaluation to be recorded and objectified could be helpful in clinical practice.

In 2009, MacDougall et al. [21] introduced an easy-to-use video system measuring eye velocity during head rotation called the video head impulse test (vHIT). In a prospective study, the horizontal VOR was recorded simultaneously with a search coil and the vHIT in 8 healthy subjects, 6 patients with vestibular neuritis, 1 patient after unilateral intratympanic gentamicin therapy and 1 patient with bilateral vestibular loss. The authors validated this new diagnostic method and reported that it is equivalent to search coils in evaluating peripheral vestibular loss. Moreover, it can be easier to use in clinics, even in patients with acute vestibular neuritis.

**Figure 2:**
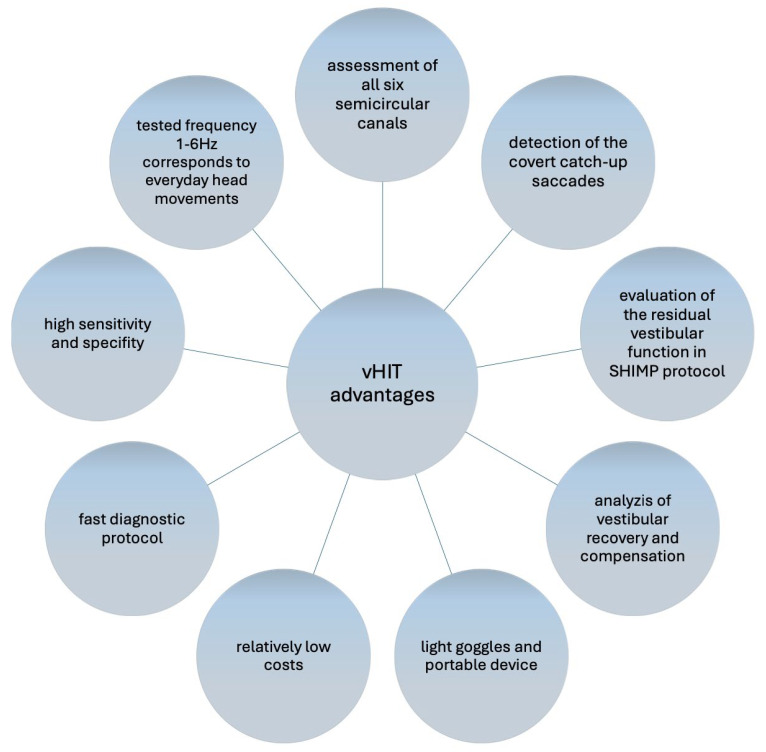
The advantages of the video head impulse test (vHIT). SHIMP—suppression head impulse paradigm.

As there are several commercial vHIT devices available and the costs are relatively low in comparison to other vestibular tests, the method is becoming more widely accessible. There are numerous advantages of this diagnostic methos (Figure 2). Nevertheless, to our best knowledge, the availability of the vHIT in the emergency department in individual countries and regions has not yet been described in the literature. Further studies analyzing the use of this method and its costs would be very valuable.

### Technical and Practical Aspects 

The examination is performed using lightweight goggles mounted on an elastic band placed on the patient’s head. The procedure should start by adjusting the band to minimize the slippage of the frame with the camera and then setting the camera so that it records the entire pupil. During the examination, the patient sits in front of a wall or screen at a distance range of approximately 1.2–1.5 m and is asked to look at one stationary point at eye level. The examiner stands behind the patient and performs fast movements of the patient’s head in the plane of the lateral semicircular canals, i.e., to the right and left. Although the examination can performed with two different examiner’s hand positions, the technique can affect the reliability of the test. The jaw hand position, in comparison to the head hand position, is considered to increase the accuracy of the vHIT in determining the side of VN [22]. The technique is similar to the clinical assessment of the HIT, and the trials should be abrupt (peak velocity range of 150–300°/s, duration range of 100–200 ms) and have a range of approximately 15–20°. When analyzing patients with VN, a high-velovity vHIT (240°/s) is considered more sensitive to a low-velocity vHIT (80°/s), with a difference range of 17–20% based on pathologic vHIT gains and the presence of the saccades [23]. Similarly to the bedside examination, the side of the movements should be random and unpredictable to avoid anticipating saccades. If the trial is performed incorrectly, the system automatically removes it and it will not be taken into account in the analysis. Usually, 10 to 20 technically correct trials are performed for each side.

The key advantage of the vHIT is the ability to evaluate all six semicircular canals. To assess the vertical angular VOR, head movements are performed in the planes defined by the pairs of tested canals, namely right anterior and left posterior (RALP) and left anterior and right posterior (LARP).

**Figure 3:**
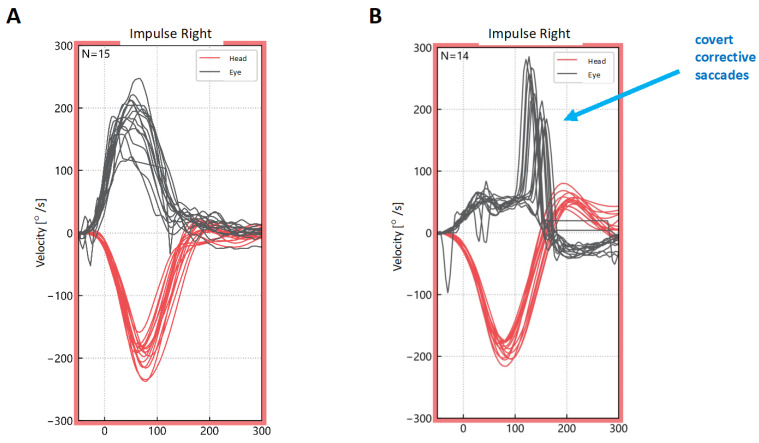
Video head impulse test (vHIT) results—lateral plane, impulse right. (**A**) Normal vHIT result with the eye movement curves corresponding to head movement curves but directed in the opposite direction. (**B**) Abnormal vHIT result in a patient with acute vestibular loss on the right side, showing reduced gain (flattened eye movement curve) and corrective saccades.

The vHIT result is a graph of the speed of the patient’s head and eye (Figure 3). when interpreting the test, the most important aspect is to analyze the graph depicting the eye velocity. If the curve for the eye movement resembles the curve of the head movement but in opposite directions, the result is considered normal (Figure 3A). In cases of vestibular loss, corrective saccades will be typical (Figure 3B). In comparison to the bedside examination, the vHIT makes it possible to detect covert saccades taking place during head movement. The second assessed parameter of the vHIT is the gain, defined as the ratio of the eye velocity to head velocity, which in a healthy patient is approximately 1 (0.8–1.2). When assessing the test results, the symmetry is also analyzed, with interaural asymmetry rates of up to 20–25% usually accepted [21]. During the acute phase of VN, overt compensatory saccades and low VOR gains are the most characteristic findings in the vHIT.

## 4. Application of the vHIT in the Diagnosis of VN

Although the diagnosis of VN is based mainly on clinical characteristics, in recent years, numerous studies have analyzed the use of vestibular tests in the diagnosis of VN. Abnormalities for the lateral canal are the most commonly described aspect of the vHIT in patients with VN, with pathological results found in 91.4 to 97.7% of patients [24,25,26,27,28,29]. To evaluate the value of the vHIT in the early diagnosis of VN, Guan et al. [27] analyzed VOR gains in 33 patients with VN and compared them with 96 patients with other types of acute vertigo (peripheral and central) and 50 healthy controls. The sensitivity in differentiating VN from other acute vertigo types or the asymptomatic control group was 87.9% and the specificity was 94.8%. Nevertheless, the results were not correlated with other vestibular tests and the study population was heterogenous, which constituted a significant limitation of that study.

### 4.1. Correlation between Caloric Tests and the vHIT in Patients with VN

Although videonystagmography (VNG) with caloric tests assessing unilateral weakness (UW) remains the gold standard in unilateral vestibular loss, the application of new diagnostic methods has been the subject of several studies in recent years [30,31,32,33,34,35,36]. Both caloric tests and the vHIT evaluate the angular VOR of the lateral semicircular canal. Nevertheless, the stimuli in these methods are different and they evaluate ampullar sensory cells at disparate frequencies; caloric irrigations test the lower frequency range of about 0.003 Hz, whereas the vHIT assesses high frequencies of 1 to 5 Hz [28]. Recently, the dissociation between VNG and vHIT results among patients with different causes of vertigo were reviewed by Waissbluth et al. [29].

According to Lee et al. [30], among 36 patients diagnosed with VN, 80.56% showed normal VOR gains in the vHIT and canal paresis with VNG, whereas in 19.44% low gains and normal caloric responses were found.

In patients with no significant asymmetry undergoing caloric tests (UW up to 25%), the bedside HIT most often does not reveal pathological corrective saccades, with a UW rate of 42.5% described as the cut-off point for the presence of abnormal HIT [31]. 

Mahringer et al. [32] analyzed the results of bedside HITs and vHITs in 172 patients complaining about vertigo or dizziness in whom UW of at least 25% was confirmed in a caloric test. A pathological vHIT result was found in 41% of the study population. Then, the group was divided depending on the duration of the symptoms, and an abnormal vHIT result was present in 63% of patients with acute vertigo (within 5 days after the symptoms onset), whereas among the non-acute group only 33% of the patients had a pathological vHIT. The specificity and sensitivity rates of the bedside HIT in the study population, compared to the vHIT, were 74% and 89%, respectively. However, in patients with acute vertigo, the sensitivity of the bedside HIT compared to the vHIT increased to 93% and the specificity was 72%. Additionally, both the bedside and video HIT results correlated significantly with the VNG, and pathological HIT results were more common in patients with higher UW values. These results are consistent with the previous study by Bartolomeo et al. [33], who reported sensitivity and specificity rates of 68.84% and 100%, respectively, at the caloric testing value of 30%. Similarly, McCaslin et al. [34] showed that the sensitivity was 78% and the specificity was 95%.

To investigate the relationship between caloric tests and vHIT results, Redondo-Martínez et al. [35] performed both examinations in 20 patients with VN and analyzed the correlations between them and with the Dizziness Handicap Inventory (DHI) for each patient at two different moments of the symptoms’ evolution. No correlation was found between the canal paresis and gain asymmetry in either the acute phase (up to 5 days after the symptoms onset) or in the follow-up (30 to 90 days later). Moreover, gain increases in the vHIT did not correlate with an improvement of the DHI result, indicating that a gain increase as the only evaluated parameter is not related to a subjective improvement of the patient’s functional level.

Several authors concluded that the VNG and vHIT should be complementary to each other, as they assess different frequencies of VOR and the vestibular stimulation is different in these methods [32,36]. Nevertheless, the pathogenesis of the dissociation of caloric tests and the vHIT in patients with acute VN remains unclear and further studies are necessary.

### 4.2. Analysis of the Corrective Saccades in Patients with VN

Catch-up saccades are the most important markers of vestibular loss visible in the bedside HIT. However, some of them occur during the patient’s head movement and are not be visible to the naked eye. Importantly, corrective saccades may co-occur with normal gain values; thus, a detailed analysis of the vHIT result should be performed in patients suspected of displaying vestibular loss [37]. 

A comprehensive analysis of the covert corrective saccades was described by Blodow et al. [38]. Among 52 patients with VH, an abnormal vHIT result, characterized by the decreased gain and corrective saccades, was present in 94.2% of cases. In that group, both overt and covert saccades were visualized in nearly half of the patients (49%) and isolated overt saccades were present in 34.7%, while in the remaining 16.3% of patients only isolated covert saccades were detected in the vHIT. Moreover, according to Redondo-Martinez et al. [35], an analysis of the catch-up saccades is crucial after the acute phase of VN symptoms, as 55% of patients can exhibit overt saccades and 65% covert saccades. Thus, there is a certain percentage of isolated covert saccades in VN that need video recordings to be confirmed.

To verify whether an analysis of the corrective saccades improves the vHIT sensitivity in the VN diagnosis, Yang et al. [39] examined 63 patients in the acute phase and at the follow-up visit one month later. Interestingly, during the acute phase, the abnormal rates based on the vHIT gain and peak velocity of catch-up saccades did not differ significantly; the percentages of abnormal results were 87% and 97%, respectively. However, at the follow-up visit, the proportions of abnormal results were significantly higher when considering both the gain and corrective saccade values than considering the vHIT gain only, at 87% and 62%, respectively.

Interestingly, some subtle abnormalities can also be detected in the vHIT examination on the unaffected side. According to a retrospective analysis performed by Manzari and Tramontaro in 2021 [40], quick anti-saccades are observed during the patient’s head movement toward the opposite asymptomatic side. These were found in all 32 patients with VN in the acute phase within the first 24 h after symptom onset; however, in the follow-up examination performed after 8 weeks, they regressed in 90.63% of patients. Nevertheless, these quick eye movements directed away from the affected side require differentiation from the quick phase of spontaneous nystagmus that can be present despite visual fixation in the acute phase of vestibular loss. 

### 4.3. SHIMP Protocol in VN Diagnostics

In addition to the standard head impulse paradigm (HIMP), it is possible to evaluate VOR suppression in the suppression head impulse paradigm (SHIMP). During that part of the vHIT examination, the patient has to look at the head-fixed target during energetic, unpredictable head movements, similarly to the HIMP protocol. Thus, in the SHIMP paradigm, the patient’s task is to follow the moving target against the VOR. As it is impossible to inhibit the VOR completely, anti-compensatory saccades in the direction of the head impulse will be visible in healthy patients’ SHIMP results, whereas vestibular loss will be evident in the results as a graph corresponding to the head movements, without saccades. Thus, anti-compensatory saccades are considered a sensitive sign of residual vestibular function. Recently, some studies reported that the SHIMP could be very useful in vestibular loss diagnoses, as the gain is not affected by the covert saccades and can be evaluated more precisely [41,42,43]. Moreover, as a possible key advantage in patients with VN, the SHIMP result is not affected by the spontaneous nystagmus, as the saccades are in the opposite direction.

To analyze the correlations between the HIMP and SHIMP protocols, Chen et al. [44] performed both vHIT paradigms in 40 patients with VN affecting the superior vestibular nerve and 20 healthy participants. The authors found a significant positive relationship between HIMP and SHIMP gains, whereas the frequency of occurrence of saccades correlated negatively between the two paradigms. Moreover, strong negative correlations were detected between HIMP and SHIMP saccade amplitudes. When analyzing the results of the SHIMP protocol precisely, the authors found positive correlations between the gain and saccade incidence rates, as well as between the gain and saccade amplitude rates. The results of that study were consistent with the previous report by Park et al. [45], who analyzed the vHIT results in 21 patients with acute peripheral vertigo within 3 days from the onset of symptoms. The VOR gains in the HIMP and SHIMP protocols correlated with each other; however, the SHIMP gain was slightly lower. In the HIMP protocol, a mean gain of 0.74 or 0.82 discriminated the affected from the healthy ears with 95% sensitivity and 91% specificity, whereas in the SHIMP test a gain of 0.68 discriminated the affected from the healthy sides with 95% sensitivity and 91% specificity. As the SHIMP gain is not affected by the velocity of the covert saccades, the authors claimed that the SHIMP can be used to reduce measurement errors of VOR gain. Moreover, the HIMP gain correlated significantly with the peak velocity of the anti-compensatory saccades in the SHIMP. Thus, the peak saccade velocity may be an additional parameter of the SHIMP used as a complement for evaluating vestibular function.

### 4.4. Diagnosis of Inferior Branch VN

VN can affect the superior, inferior or both branches of the vestibular nerve. The superior vestibular nerve is considered more likely to be affected due to the anatomical conditions; the bony channel of the superior branch is seven times longer and is narrower [24,46,47,48,49]. It is known that the superior vestibular nerve innervates the anterior and lateral semicircular canals, while the inferior branch innervates the posterior semicircular canal. In the bedside examination, isolated superior vestibular nerve involvement can manifest as horizontal–tortional nystagmus in a plane corresponding to the lateral and anterior semicircular canals, according to the Ewald’s first law [50]. However, as the horizontal component is more intense, it is not possible to precisely assess canal involvement with the naked eye. 

Thanks to the advancement of the vHIT technique, it has become possible to evaluate each semicircular canal. According to Zhang et al. [51], who correlated the characteristics of nystagmus recorded via VNG with vHIT results in patients with VN, there is a correlation between the direction and slow phase velocity characteristics of the nystagmus and the VOR gain of each semicircular canal. 

According to Psillas et al. [52], who analyzed the vHIT results in 35 cases of VN examined within the first 10 days after symptom onset, the majority of patients (80%) in the acute stage of VN had lower VOR gain values in the lateral and anterior semicircular canals compared to the posterior semicircular canal on the affected side (48.5%). 

Büki et al. [53] analyzed the vHIT results in 44 patients with acute vertigo and diagnosed superior VN affecting the anterior and lateral semicircular canals in 43% of patients, whereas isolated posterior canal dysfunction was observed in 9.1% of cases only. 

To characterize the profiles of vestibular end organ dysfunction, Taylor et al. [54] analyzed a set of otoneurological tests including the vHIT and ocular and cervical vestibular evoked myogenic potentials (oVEMPs and cVEMPs) in 43 patients with acute VN. When analyzing the vHIT results, abnormal VOR gains of the lateral and anterior semicircular canals were found in 97.7% and 90.7% of patients, respectively, whereas dysfunction of the posterior canal was significantly less frequently observed (39.5%). However, when taking into consideration both the vHIT and VEMPs, 55.8% of patients had abnormalities localized to both vestibular nerve branches, as there were cases with dysfunction of the lateral or anterior semicircular canal with an abnormal cVEMP result, indicating saccule dysfunction. Isolated inferior VN was found in one patient only (2.3% of the study population). 

In summary, the vHIT can be used to objectify the involvement of individual semicircular canals in patients with VN. The results of numerous studies confirm that the superior vestibular nerve is affected more often than the inferior branch. 

### 4.5. Compensation and Recovery Patterns in vHIT

The clinical manifestations of VN vary depending on the time since the symptoms started. After a period of acute vertigo, the symptoms gradually subside as the peripheral and central compensation start. As the static compensation occurs, withdrawal of the spontaneous nystagmus and catch-up saccades is visible in the bedside examination. In precise and objective analyses of the angular VOR during the VN’s natural course, a vHIT could be helpful. 

After the acute phase of VN, the vestibular function may return to normal, which will be visible as an increase in the gain and withdrawal of the saccades in the vHIT. However, in some patients, restoration of the semicircular canals’ function does not occur despite clinical recovery, and covert catch-up saccades are considered to be sufficient to successfully compensate the vestibular loss [55,56].

**Figure 4:**
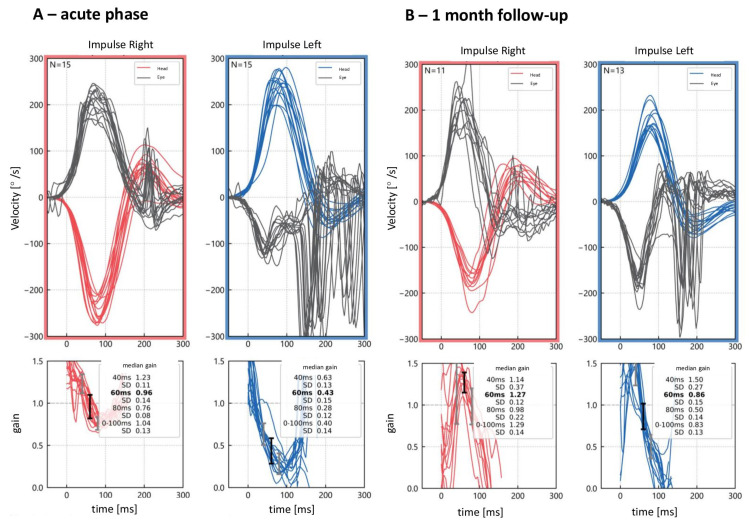
Video head impulse test (vHIT) results in a patient with vestibular loss on the left side. (**A**) Examination performed in the acute phase two days after the symptoms’ onset, showing numerous overt and covert saccades and a gain reduction (0.43). (**B**) Follow-up examination 1 month later, showing changes of the organization of the saccades, with a prevalence of covert saccades and increase in the gain value to normal (0.86).

Depending on the stage of VN, different patterns of VOR can be distinguished in the examination (Figure 4). In a retrospective study by Manzari et al. [55], patients were divided into two subpopulations according to the time since the acute vertigo occurred. Significant differences were reported between patients in the acute (within the first 72 h) and subacute (4 days to 6 weeks after the symptoms onset) stages, with the horizontal VOR gain being analyzed. Clinically, the two groups differed in their severity of symptoms assessed in the DHI. Moreover, a significant correlation between the VOR gain and subjective functional level was found after the acute phase of VN.

To analyze the recovery pattern of VOR, Fu et al. [56] examined 47 patients with VN within 10 days of symptom onset and repeated the test after about 6 months. At the follow-up, significant VOR gain recovery on the lesion side was found. Moreover, reductions in the incidence and peak velocity of the overt and covert saccades was observed. To analyze the relationship between the vHIT result and functional compensation after VN, at the follow-up visit the patients were divided into two groups, namely a normal to mild dizziness group (DHI score ≤ 30) and moderate to severe handicap group (DHI score > 30). Interestingly, the mean gain in the first group was significantly higher than in the second group; moreover, the patients with normal to mild dizziness exhibited a significantly higher normal rate of VOR gain at the follow-up visit. When analyzing the saccade patterns, high occurrence rates of isolated covert saccades and lower velocities of covert saccades were characteristic in the normal to mild dizziness group. In summary, both gain recovery resulting from the peripheral sensory cells’ regeneration and a change in morphology of the corrective saccades can occur during the vestibular compensation. Presumably, in patients with persistent decreased gain, fast covert saccades taking place during the head movement can be crucial to compensate for the slow-phase eye velocity in daily life. An important role of covert corrective saccades is confirmed by their correlation with a patient’s subjective functional level.

Typically, the symptoms of VN last days or weeks, although half of the patients experience chronic dizziness and imbalance [57,58]. To better understand the underlying pathophysiology causing the incomplete balance compensation, a comprehensive vestibular test set repeated at various stages of VN is needed. Navari et al. [49] verified whether the vHIT can be useful in evaluating the efficacy of the vestibular rehabilitation. They analyzed the clinical symptoms and vHIT results before and after rehabilitation in 30 patients with a past history of VN complaining about residual balance symptoms. After 10 weeks of vestibular rehabilitation, all patients reported a subjective improvement, which was consistent with the DHI scores. In the follow-up, a significant improvement of the VOR gain and reduction in gain asymmetry were found. Interestingly, despite a significant reduction in the number and amplitude of overt corrective saccades, no change was observed in terms of the covert saccades. These results show that repeated vHIT examinations can be potentially useful in the follow-up after treatment [59]. This observation of the persistent covert catch-up saccades indicates their role in dynamic visual acuity compensation, which is consistent with the aforementioned study [55].

The amplitude and latency of the corrective saccades, as well as their organization and grouping, can be analyzed in patients after VN. Moreover, the organization of the saccades in a gathered pattern can be induced in patients without spontaneous compensation [60].

Taking advantage of the unique opportunity to evaluate the function of the vertical semicircular canals, Patel et al. [61] analyzed angular VOR gains in all planes and their correlations with subjective functional improvements 3 months after VN. Among 20 patients, 60% reported subjective recovery, while the remaining 40% of the study population complained of persistent balance problems. Interestingly, no significant differences in vHIT gain were found between these groups and no correlation between the DHI scores and VOR gain or vHIT gain asymmetry was found. 

To characterize changes occurring after the acute phase of VN in all semicircular canals, Psillas et al. [52] examined 35 patients within the first 10 days of symptoms and repeated the test at the follow-up visit 6–30 months later. The results were compared to a group of 32 healthy volunteers. In the majority of VN cases, abnormal vHIT results were found in all semicircular canals on the affected side. However, in the acute phase, gain reductions and corrective saccades were more prevalent in the lateral semicircular canal compared to the vertical canals. At the follow-up visit, the authors found that the gain was best recovered in the anterior compared to the lateral and posterior semicircular canals (50%, 42.8% and 41.1%, respectively). The authors concluded that the compensation occurs more efficiently in the superior vestibular nerve and mainly involves the horizontal canal, as the covert saccades were more frequently found in that canal. Different results regarding the recovery pattern were presented by Büki et al. [53], who showed that normal gain occurred more often in the lateral semicircular canal (55%) compared to the anterior (38%) and posterior (38%) canals, indicating that the vertical canals recover less effectively. Moreover, they presumed that isolated inferior VN can be related to a worse compensation prognosis.

As presented by Lee and Kim [62], some SHIMP parameters can also be used in evaluations of recovery in patients with VN. The authors reported that anti-compensatory saccades in the SHIMP might be correlated with incomplete vestibular compensation. As they are not found during vestibular compensation in the recovery phase of VN, they may be potential indicators of the risk of chronic balance symptoms. Casani et al. [63] divided patients with a history of VN into two groups—patients who recovered spontaneously and a group with persistent balance problems. At the follow-up visit 4–8 weeks after the symptoms’ onset, the SHIMP gain was statistically correlated with the DHI result. The authors described a characteristic pattern of vHIT results in patients with incomplete compensation; beyond the lower gain values and numerous saccades in the HIMP protocol, decreased gain and lower gain prevalence rates in the SHIMP protocol were found in the follow-up examination.

In the evaluation of the vestibular compensation process, the clinical outcome and patient’s functional level remain the most important factors. Nevertheless, a battery of vestibular tests performed in the acute phase and repeated during the follow-up may provide important information about the physiology of the peripheral and central compensation. The application of the vHIT offers a unique opportunity to assess the function of all semicircular canals during the acute phase and recovery in patients with VN.

### 4.6. vHIT in the Differential Diagnosis of VN

In patients with acute vertigo episodes, an accurate diagnosis and differentiation between the peripheral and central pathologies is crucial and should be made as quickly as possible. In comparison to other vestibular tests, the vHIT can be easily performed in the emergency department, as the device is light, portable and relatively cheap. Moreover, the examination lasts only a few minutes, which makes it significantly faster than VNG. 

Apart from the vestibular causes, in 3–25% of patients, the acute dizziness may have an ischemic origin affecting the cerebellum or brainstem [64,65,66]. Due to the similar clinical manifestations, some patients with acute dizziness are misdiagnosed. Using the HINTS protocol, a preliminary diagnosis usually can be made in the emergency department without additional tests. Among the HINTS measures, the HIT is considered to have the highest sensitivity [14]. However, an abnormal bedside HIT result can be present in 9–39% of patients with acute posterior circulation strokes [13,14,15,16,67]. Thus, whereas the normal HIT is strongly suggestive of a central pathology, corrective saccades can be visible in that group of patients as well. Moreover, several studies reported that the sensitivity of the bedside otoneurological examination depends on the clinician’s skills; thus, researchers are looking for methods that enable objective and rapid VOR assessments. To verify that hypothesis, Machner et al. [17] analyzed the medical records of 38 patients who came to the emergency department with an acute vertigo episode. They reported that a bedside HIT performed by the clinicians in the emergency department had high sensitivity for VN (88%), although a false abnormal HIT result was described in 36% of patients with posterior circulation stroke; thus, the specificity was low (64%). As a false pathological bedside HIT result can lead to a misdiagnosis of stroke patients, the authors recommend the vHIT as an additional diagnostic test when a specialist trained in otoneurological examination is not available in the emergency department. 

According to the prospective observational study published by Thomas et al. [68], who examined 133 patients with acute vertigo episodes, the vHIT can improve the sensitivity of the HINTS examination and help to exclude a central etiology of acute vertigo. The authors reported that the standard HINTS had a sensitivity of 83% and specificity of 86%, whereas the ‘v-HINTS’ (HINTS examination with the vHIT substituted for the bedside HIT) was superior to the standard HINTS, with a sensitivity of 89% and a specificity of 96% for the diagnosis of VN. 

To assess the diagnostic efficacy of the vHIT in differentiating VN and posterior circulation stroke, Ha et al. [69] analyzed VOR gains in the vHIT in 29 patients with VN and compared them with 40 cases of dorsal brainstem stroke. Their detailed analysis showed that the VOR gain in the ipsilesional lateral canals was significantly lower in patients with VN compared with the group diagnosed with brainstem stroke, and confirmed that the vHIT can effectively differentiate between central and peripheral acute dizziness. Similarly, Nham et al. [70] reported that the lateral vHIT gain on the affected side was lower in patients with VN than stroke. Moreover, they observed that corrective saccades were visible more often in patients with VN, and their amplitude and velocity were greater in VN than in patients with posterior circulation stroke.

Guler et al. [71] used a gain cut-off of ≤0.75 to analyze the vHIT’s sensitivity in differentiating between peripheral and central vertigo. The authors reported that 96% of the patients with acute vestibular loss could be diagnosed with a specificity of 93% using that protocol. However, 44% of patients with AICA–PICA ischemia also had lowered gains of ≤0.75. Moreover, the authors reported that adding a gain asymmetry rate of ≥17% to the gain cut-off allowed them to achieve higher diagnostic accuracy, with 100% of posterior fossa ischemia cases correctly diagnosed.

Importantly, normal VOR gain is often found in patients with PICA stroke, although it is not typical for AICA ischemic lesions [72]. Although the VOR gains differ between peripheral and central causes of vertigo, AICA strokes can be misdiagnosed based on the VOR gain alone [71,72]. Thus, a comprehensive evaluation of the vHIT including a corrective saccade analysis may be crucial. As reported by Calic et al. [73], a gain value >0.68 separated VN from posterior fossa strokes, with a sensitivity of 95.5% and specificity of 68.2%, while combining the VOR gain and saccade prevalence rates allowed them to separate the peripheral and central vertigo cases in 90.9% of patients.

Interestingly, a recent study suggested that artificial intelligence (AI) applied in MRI and vHIT analyses can also be helpful in differentiating between peripheral vestibulopathies and central ischemic causes of vertigo [74].

## 5. Conclusions

Acute vertigo or dizziness is reported by up to 7% of patients presenting to the emergency department. Thus, it should be considered as a multidisciplinary diagnostic problem that neurologists, otorhinolaryngologists and emergency department doctors deal with. Despite numerous studies having been performed, the etiology of VN remains unclear, making treatment and follow-up in this group of patients difficult. Moreover, the central pathologies can mimic peripheral vestibular loss, which is called vestibular pseudoneuritis. This can lead to the inappropriate management and mistreatment of stroke patients requiring immediate therapy. As a portable and easy diagnostic tool enabling the VOR to be recorded and objectified, the vHIT can be helpful in patients with acute vertigo in the emergency department. Moreover, it can be used during follow-up to evaluate their recovery and compensation.

## Data Availability

No new data were created or analyzed in this study.
